# Fracture Toughness and Fatigue Crack Growth Analyses on a Biomedical Ti-27Nb Alloy under Constant Amplitude Loading Using Extended Finite Element Modelling

**DOI:** 10.3390/ma16124467

**Published:** 2023-06-19

**Authors:** Mohammed Y. Abdellah, Hamzah Alharthi

**Affiliations:** 1 Mechanical Engineering Department, Faculty of Engineering, South Valley University, Qena 83523, Egypt; 2 Mechanical Engineering Department, College of Engineering and Islamic Architecture, Umm Al-Qura University, Makkah 24382, Saudi Arabia

**Keywords:** implant, biomaterial, bone, XFEM, J-integral, fracture toughness

## Abstract

The human body normally uses alternative materials such as implants to replace injured or damaged bone. Fatigue fracture is a common and serious type of damage in implant materials. Therefore, a deep understanding and estimation or prediction of such loading modes, which are influenced by many factors, is of great importance and attractiveness. In this study, the fracture toughness of Ti-27Nb, a well-known implant titanium alloy biomaterial, was simulated using an advanced finite element subroutine. Furthermore, a robust direct cyclic finite element fatigue model based on a fatigue failure criterion derived from Paris’ law is used in conjunction with an advanced finite element model to estimate the initiation of fatigue crack growth in such materials under ambient conditions. The R-curve was fully predicted, yielding a minimum percent error of less than 2% for fracture toughness and less than 5% for fracture separation energy. This provides a valuable technique and data for fracture and fatigue performance of such bio-implant materials. Fatigue crack growth was predicted with a minimum percent difference of less than nine for compact tensile test standard specimens. The shape and mode of material behaviour have a significant effect on the Paris law constant. The fracture modes showed that the crack path is in two directions. The finite element direct cycle fatigue method was recommended to determine the fatigue crack growth of biomaterials.

## 1. Introduction

Fatigue fracture is the most common type of damage in implants used in the human body to replace injured bone [1]. In addition, implants used in bone replacement are subjected to cyclic loading conditions [1]. In biomedicine, many types of materials are used as implants: stainless steel, titanium and its alloys, and Co-Cr materials. In addition, biomaterials can be natural or artificial materials that are commonly used as implants in humans to replace injured or lost bones to enhance the comfort of life of the individual or patient [2,3,4,5]. Titanium and its alloys are given special consideration due to their good biocompatibility and biodegradability [6,7]. Titanium alloys such as Ti-6Al-4V, a commonly known biomedical alloy, are characterised by a high fatigue strength of 460 MPa when tested at room temperature and a high frequency of 20 kHz. Furthermore, the stress amplitude decreases with an increasing number of cycles [8]. Titanium alloys used as implants are usually subject to low cycle fatigue [9].

The fatigue crack growth FCG, other mechanical properties, and biocompatibility of titanium and its alloy are highly influenced by surface treatment [9,10,11]. In addition, the microstructure of titanium alloy has a great influence on fatigue crack growth, as the grain size and its arrangement affect crack growth behaviour [12,13,14]. Moreover, Ti-6Al-7Nb and Ti-6Al-4V showed a significant improvement in fatigue and corrosion behaviour when their surfaces were treated with titanium dioxide nanotubes [15]. Niinomi [16] investigated the mechanical compatibility properties of titanium alloys. These properties were the modulus of elasticity, wear properties, fatigue crack growth, friction fatigue, fracture toughness, and ductility. Additionally, the compressive fatigue behaviour of titanium alloys for biomedical applications was investigated. Compression fatigue was associated with commutation loading and resulted in higher fatigue strength. An early study attempted to find a correlation between the fracture toughness of titanium and its alloy and tensile behaviour. Simple correction was better for Ti with a limited range of microstructures, whereas complex corrections were valid for Ti with a wider range of microstructures [17]. Moreover, the FCG for Ti-6Al-4V was a function of crack length, as the short fatigue was determined by the cyclic stress range, while the long fatigue crack was calculated by the stress intensity factor range [18]. In a study by Prasad et al. [19], two temperatures were tested: 450 °C and 600 °C. It was found that 450 °C gave higher fatigue strength due to dynamic stretch ageing. Other studies by Wang et al. [20] investigated the effect of ageing temperature and times or their types [21] on the mechanical and microstructural properties of Ti-Nb alloys for biomedical applications. It was found that higher ageing temperature increases tensile strength, improves ductility, and increases plasticity.

On the other side of the study, a numerical model was implemented to predict the mechanical behaviour of biomedical material in order to avoid expensive and destructive mechanical characterisations. Neto et al. [22] numerically investigated the FCG for the alloy Ti-6Al-4V. The simulation was performed for a compact tensile test, and the effects of crack blunting, material hardening, and crack closure problems were also considered, which were the main reason for the overload results in the FCG behaviour. In addition, a more advanced FE modelling to predict the FCG, tensile strength, and fracture toughness of Ti-6Al-4V alloy was prepared by Verma et al. [23]. MATLAB was used to extract the XFEM equations using the Abaqus package. The model showed that the FCG was higher at a high stress ratio. Additionally, the MATLAB XFEM was available to predict the fracture behaviour and FCG. The FCG increased at lower stress ratios, while the fracture toughness in the centre of the crack surface was predicted to be critical in the FCG case.

A titanium alloy, Ti-27Nb, has played a dominant role in biomedical applications as an attractive bi-material for implants and other bone substitutes due to its good fatigue resistance, non-toxicity, and good biocompatibility with good mechanical properties [24,25]. It has lower ductility but higher fatigue strength, which makes it more suitable for cyclic loading applications such as implants [26,27,28]. In addition, the fracture toughness of the Ti-27Nb alloy was 50 $\mathrm{MPa}\sqrt{m}$, which was less than the other known biomedical titanium alloy Ti-6Al-4V, which was 65 $\mathrm{MPa}\sqrt{m}$ [29]. The fatigue fracture behaviour of the Ti-27Nb alloy showed brittle fracture with transgranular and intergranular fracture patterns as reported in [29,30]. An older study by Amjad et al. [31] looked at the Ti-27Nb alloy and simulated the effects of daily life on the performance of a human implant made from this alloy.

The Ti-27Nb alloy is a relatively new material and a promising material for biomedical applications, as shown in the previous review. The fatigue properties and fatigue crack growth need to be studied in more detail for this type of material, in particular, numerical studies and robust mathematical tools to predict the occurrence of fatigue cracks. Therefore, the present work focuses on the following objectives: (1) extraction of a finite element model FEM to predict the R-curve of the Ti-27Nb alloy at room temperature, (2) comparison of the results obtained by other standard forms of fracture toughness measurement based on a single value called J-integral, and (3) derivation of a FEM to predict the FCG under constant amplitude in the low cycle fatigue region and comparison of the failure modes published with other available results.

In Section 1, the properties of Ti-27Nb alloy are outlined; in Section 2, the fracture toughness calculated by XFEM is extracted, followed by the method to draw the R-curve; and in Section 3, the FCG analysis using XFEM is discussed and derived. Finally, a comprehensive comparison is performed to identify the best results and quantify the accuracy of the modelling method.

## 2. Material (Ti-27Nb Alloy)

**Figure 1:**
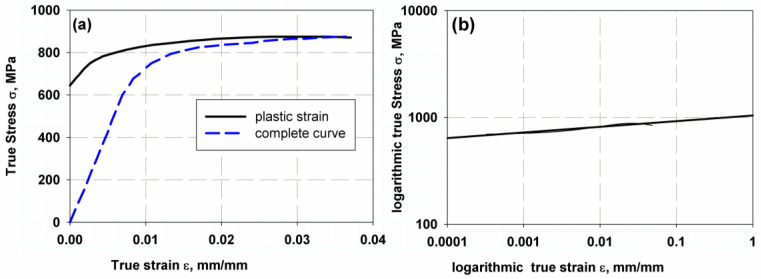
True stress–True strain relation of Ti-27Nb alloy, (**a**) Linear relation, (**b**) Log–Log relation.

The Ti-27Nb alloy has the flow properties shown in Figure 1a and listed in Table 1. The mechanical properties were determined using a simple tensile test described in Ref. [29], and the hardening properties were determined using the logarithmic scale of the yield curve (Figure 1b). These types of alloys have attractive applications in human implants, where they exhibit good biocompatibility and high durability. The main component of the Ti-2Nb alloy is titanium (Ti) and niobium (Nb), with 26.01 ± 1.05 wt% each [29]. The durability of the implant is more dependent on the fatigue life; therefore, it is important to study the behaviour of such a bioactive alloy under fatigue.

## 3. Extended Finite Element Method XFEM

The extended finite element method is a fairly new advanced method in which crack initiation and propagation are independent of the mesh. It was developed by Belychko and Blak [32] following the older model of Melenk and Babuska [33]. The method was characterised by the possibility to simulate any crack propagation without a mesh [34]. Therefore, the problems of calculation of fracture toughness and stress intensity factor could be solved in a short time with very accurate results [35]. The mesh-free function (Equation (1)) of crack propagation by XFEM is as follows [34]:

(1) $$u^{h}=\underset{i\in I}{\sum }u_{i}N_{i}\left(x\right)+\underset{i\in I}{\sum }a_{i}N_{i}H\left(x\right)+\underset{i\in k1}{\sum }N_{i}\left(x\right)\left(\overset{4}{\underset{l=1}{\sum }}b_{i,1}^{l}F_{1}^{l}\left(x\right)\right)+\underset{i\in k2}{\sum }N_{i}\left(x\right)\left(\overset{4}{\underset{l=1}{\sum }}b_{i,1}^{l}F_{2}^{l}\left(x\right)\right)$$

where $u_{i}$ is the displacement unit, l is the nod at the element, $N_{i}$ is the geometric function of node i, $a_{i}$ is the crack length, $H\left(x\right)$ is the Heaviside function at the subset of enriched nodes (i∈I), (i∈k1), and (i∈k2) are the set of nodes to enrich to model crack tips numbered 1 and 2, respectively. $b_{i,1}^{l}$, $b_{i,2}^{l}$ are displacements of nodes 1 and 2. The Heaviside function ($H\left(x\right)$) is defined as follows:

(2) $$H\left(X\right)=\left\{\begin{array}{c} -1,\;\;\;if\;x>0\\ 1,\;\;\;if\;x<0 \end{array}\right.$$

The use of coherent units in XFEM simulations can lead to unreliable results due to the interface effects described by the coherent interface model [36]. These effects can cause oscillations in the results and reduce the accuracy of the simulation [37]. Furthermore, the use of cohesive elements with force correction has been shown to improve the accuracy of XFEM simulations with coherent units [38]. However, more research is needed to develop effective solutions to improve the reliability of XFEM results with coherent units.

In summary, the XFEM method is a powerful numerical technique for simulating crack propagation. However, the use of coherent units can limit the reliability of the results due to interface effects. Possible solutions to improve the accuracy of XFEM simulations with coherent units include the use of cohesive elements with force correction. Further research is needed to develop more effective solutions to improve the reliability of XFEM results with coherent units [39,40]. Therefore, in the present study, the coherent surfaces were not applicable, and they let the crack propagate through the element or through boundaries.

Fracture Toughness (XFEM)

According to the LEFM theories, the fracture toughness K_IC_ or the corresponding surface release energy ($G_{IC}$), the J-integral, is well considered around the head of the crack tip. The asymptotic crack-tip functions $F_{2}^{l}\left(x\right)$ can be calculated using Equation (3) as follows:

(3) $$F_{2}^{l}\left(x\right)=\left\{\sqrt{r}\sin\left(\frac{\theta }{2}\right),\sqrt{r}\cos\left(\frac{\theta }{2}\right),\;\sqrt{r}\sin\left(\frac{\theta }{2}\right)\sin\left(\theta \right),\;\sqrt{r}\cos\left(\frac{\theta }{2}\right)\sin\theta \;\right\}$$

where (θ; r) is a polar coordinate system where the origin at the crack tip with its tangential occurs when (θ = 0), and the parameter ($\sqrt{r}\sin\frac{\theta }{2}$) considers the discontinuity across the crack face. This function has a lot of applications, including biomaterial and elastic–plastic power law hardening material.

The purpose of these functions is to calculate the fracture toughness using the J integral values and the corresponding stress intensity factor. The finite element damage model implemented to simulate the fracture toughness of Ti-27Nb alloy is based on the theory of maximum principal stress $\sigma_{1}$ at which the crack propagates. Two finite element models were implemented: the linear (elastic) behaviour through a compact tensile specimen and the nonlinear (elastic–plastic) behaviour through a tensile specimen with a central notch.

The two models were used to compare the accuracy of the specimen standards by simulating the finite element numerical model. For the linear-elastic behaviour $\sigma_{1}$ model, the value of yield strength was chosen to be 600.5 MPa. For the elastic–plastic model, a value of 874.63 MPa was chosen. In elastic or linear behaviour, the crack starts at the yield point, the critical J-integral or release energy is based on a single value at the peak load, and no plastic region is implemented. On the other hand, in elastic–plastic or nonlinear behaviour, the material with strain hardening (see Figure 1b) gives a plastic damage surface, while the crack propagates when the yield point is reached since the cohesive stress above the crack tip is considered. Therefore, the nonlinear model is used to predict the R-curve of the material at hand.

**Figure 2:**
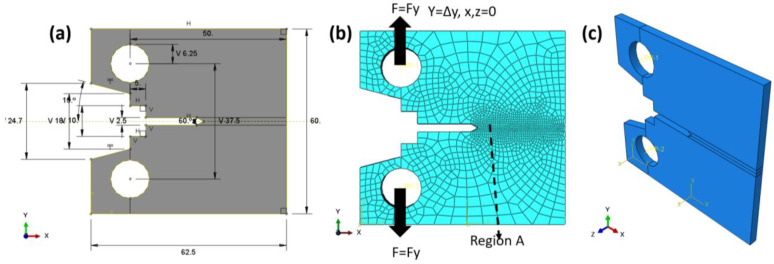
Linear FE model (**a**) CT domain, (**b**) Mesh and B. C Domain, (**c**) 3D domain with thickness.

The finite element domain of the compact tensile specimen (CT) with linear model test is shown in Figure 2a; the dimensions are in accordance with ASTM E399 [41], and the specimen is loaded in tension until failure. The boundary conditions are explained according to the experimental data in Ref. [29]; the two loaded pins were loaded with a peak load PQ of 3030 N, and the initial value per crack was 1.3 mm. No DOF was allowed across the *z*-axis or *x*-axis, and one degree of freedom was allowed across the *y*-axis (see Figure 2b). XFEM was used to measure the J-integral around the crack tip, so no crack-enriched function was determined. The total number of elements was 40,000, with a hexagonal shape, and a linear brick with eight nodes. A reduced integration and hourglass control (C3D8R) was chosen. Region A is denser and finer than the other section of the sample. The convergence of the mesh was tested many times to select these types and the number of elements.

**Figure 3:**
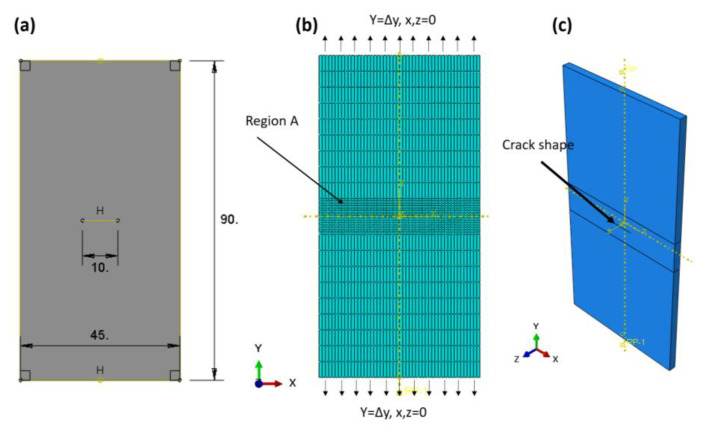
Nonlinear FEM (**a**) CNT domain, (**b**) CNT mesh and B. C domain, (**c**) 3D domain with thickness.

On the other hand, the mean notch stress (CNT) was simulated using nonlinear behaviours to progress the crack by plastic deformation and predict the R-curve of the Ti-27Nb alloy. The CNT specimen was selected according to the ASTM E-647 standard [42] and the recommendation of Newman and Haines [43] (see Figure 3), where the length-to-width ratio (L/W) is equal to 0.5 and was used many years ago as a reference for uniformly displaced and loaded specimens, which are very close to the values of stress intensity factors. The material used for the specimen was the yield curve data shown in Figure 1a and listed in Table 1. The specimen has one degree of freedom in the *y*-axis, while it is constrained in all directions of the x- and z-axes. A total of 1650 elements with the same CT characteristics and shape were used. In order to reduce the computation time, the region (A) was made denser and finer than the other regions of the samples (see Figure 3b). The model allowed us to measure the peak load (PQ) and displacement relationship during the crack propagation. According to Feddersen [44], the critical stress intensity factor can be calculated with Equation (4) as follows:

(4) $$K_{IC}=s\sqrt{\pi a}\;F\left(a/w\right)$$

where $S=\frac{PQ}{\begin{array}{c} Aremote \end{array}}$ was the remote stress over remote area Aremote, a was the crack length (10 mm), and $F\left(a/w\right)$ was a geometric correction factor related to specimen width w, which can be measure using Equation (5) [43]:

(5) $$F\left(a/w\right)=\sqrt{\sec\left(\frac{\pi a}{w}\right)}$$

Then, the corresponding surface release energy can be measured using the following Equation (6):

(6) $$G_{IC}=\frac{K_{IC}{}^{2}\left(1-\nu^{2}\right)}{E\;}$$

where E is the young modulus and ν is Poisson’s ratio.

The damage evolution law implemented in fracture toughness perdition is the maximum crack opening displacement $\delta_{Cr}$, which can be calculated using Hahn and Rosenfield’s [45] or Perez’s [46] model as follows:

(7) $$\delta_{Cr}=B\times \varepsilon_{f}$$

B is the specimen thickness = 3 mm and $\;\varepsilon_{f}$ is the fracture strain in a simple tension test.

## 4. Direct Cycle Fatigue

Direct cycle fatigue is characterised by a high stress amplitude and a low frequency, and it is characterised by a low number of fatigue cycles between 10 and 100,000 cycles and a low fatigue life [47]. In low cycle fatigue (LCF), stress is responsible for both elastic and plastic strains. Plastic strain is particularly intense in geometries with stress increases or discontinuities [48]. High-cycle fatigue, on the other hand, involves a higher number of cycles in excess of 100,000 cycles where plastic strain does not occur [47].

**Figure 4:**
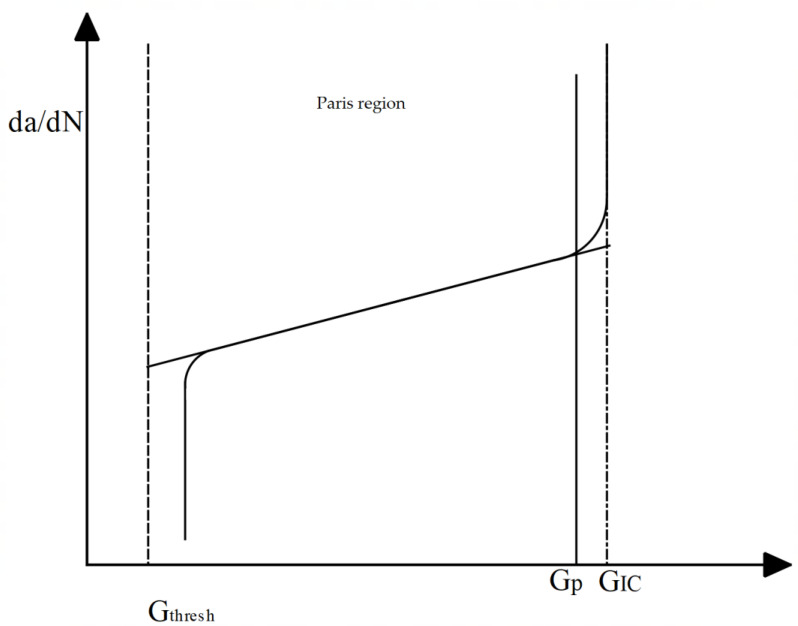
Paris’ law for fatigue crack growth.

Crack growth was controlled using Paris’ law, combining the fracture energy release rate with the crack growth rate (see Figure 4). There were two limits; the first limit was the threshold energy release rate, $G_{Thresh}$, at which no fatigue crack initiation or propagation occurs, and the second was the upper limit energy release rate, $G_{pl}$, at which fatigue crack growth propagates at an accelerated rate. The limits are normalized using the critical fracture energy release rate $G_{Ic}$ as the ratio of $G_{Thresh}/G_{Ic}$ and the ratio of $G_{pl}/G_{Ic}$, with default values of 0.01 and 0.85 chosen for these two limits, respectively.

The fatigue crack growth initiation criterion is applied at the crack tip through the interfaces, the analysis in case of law cycle fatigue based on the change of fracture energy release rate ∆G, which is defined as the difference between maximum and minimum values of fracture energy release rate [49]. The initiation criterion (see Equation (8)) implemented was as follows:

(8) $$f=\frac{N}{c_{1}∆G^{c_{2}}}\geq 1$$

where $c_{1}$ and $c_{2}$ are material constants and N is the number of cycles. The element at the crack tip fractured only after this criterion was complete and the maximum fracture energy was greater than energy $G_{thresh}$. In the present study, the value was taken as default values.

After crack initiation criterion take place and the first element at the crack tip is released, the fatigue crack growth propagation would be followed based on the fracture toughness (∆K), as in the Paris–Erdogan equation [49] shown in Equation (9):

(9) $$\frac{da}{dN}=c\;{\left(∆K\right)}^{m}$$

The XEFM was used as the fracture release energy rate *G*; therefore, using it in relation to Equation (6), the Paris–Erdogan equation can be rewritten as follows:

(10) $$\frac{da}{dN}=c_{3}\;{\left(∆G\right)}^{c_{4}}$$

where $c_{4}=\frac{m}{2}$ and $c_{3}$ can be calculated using the following equation:

(11) $$c_{3}=\frac{cK^{m}}{{\left(\frac{K^{2}}{E}\right)}^{m/2}}\;$$

The material constant values *m* and *c* were 2.9 and 10 × 10^−7^, respectively, for the Ti-27Nb alloy. These values were determined using the non-direct XFEM, whereas $c_{3}$ and $c_{4}$ were calculated as 5.226 × 10^−5^ and 1.4, respectively.

The crack growth rate was calculated from an advanced crack of the progressing path, as follows:

(12) $$\frac{da}{dN}=\frac{a_{i+1}-a_{1}}{N_{i+1}-N_{1}}$$

where i is the point of the advanced crack equal to 1, 2, or 3.

The fracture energy release rate implemented in the present study used the power law for a mixed mode of failure, as follows in Equation (13) [48]:

(13) $$\frac{G_{equiv}}{G_{equivc}}={\left(\frac{G_{I}}{G_{IC}}\right)}^{am}+{\left(\frac{G_{II}}{G_{IIC}}\right)}^{an}+{\left(\frac{G_{III}}{G_{IIIC}}\right)}^{ao}$$

The value of the mixed-mode fracture energies for the Ti-27Nb alloy is considered to be an isotropic material biocompatible with human implants and was therefore chosen to be the static fracture release energy in mode *I* at 25 kJ/m^2^.

The crack length used to calculate the fracture toughness is a two-point midpoint linear fit (average length of two adjacent points) [50,51]. Therefore, the change in fracture toughness (∆K) can be calculated in Equation (14), as follows [52]:

(14) $$∆K=K_{max}-K_{min}$$

For low-cycle fatigue with a higher stress ratio, the relation between stress ratio R with the fracture toughness ratio was as follows [53]:

(15) $$R=\frac{K_{min}}{K_{max}}$$

By instituting Equation (15) into Equation (14), an expression for the change of fracture toughness can be seen, as shown in Equation (16).

(16) $$∆K=K_{max}\left(1-R\right)$$

### Mesh Convergence and Mesh Refinement

One of the major advantages of using the extended finite element method (XFEM) is its reduced dependence on mesh convergence studies. Mesh convergence studies are typically used in a finite element analysis (FEA) to ensure that the model accurately captures the behaviour of the system being analysed [54,55]. However, mesh refinement can also increase the time and cost of analysis, and may not always provide an accurate solution for certain problems [56,57]. With XFEM, the need for extensive mesh convergence studies is greatly reduced, as the method is designed to handle discontinuities and singularities in the solution without requiring a fine mesh [57]. This not only saves time and resources but also allows for more accurate results with less computational effort.

Another advantage of using XFEM is the improved accuracy that can be achieved with coarser meshes. Traditional FEA methods require a fine mesh to accurately capture discontinuities or singularities in the solution [58]. However, XFEM can accurately model these features with a coarser mesh, which reduces the computational cost and time required for analysis [59]. This makes XFEM an attractive option for problems where a fine mesh may be difficult to create or computationally expensive to solve.

XFEM also offers significant time and cost savings in simulation studies. With XFEM, there is no need for remeshing as the geometry of the fracture does not depend on the mesh structure [57]. This eliminates the need for costly and time-consuming remeshing procedures, which can be a significant bottleneck in simulation studies. Additionally, XFEM has been automated to arrive at a convergent mesh, which further reduces the time and cost of analysis [60]. Thus, XFEM offers a powerful and efficient tool for modelling discontinuities and singularities in the solution, while also providing significant time and cost savings in simulation studies.

When damage occurs in ductile metal, the relationship between stress and strain is strongly dependent on mesh size and density. Therefore, a damage assessment law was derived by Hillerborg [61] based on a material parameter called fracture energy, $G_{f}$, which is energy used to separate crack length units. The fracture toughness is expressed in terms of the characteristic length to reduce the dependence on mesh size, as follows:

(17) $$G_{f}=\int_{{\bar{\epsilon }}_{0}^{pl}}^{{\bar{\epsilon }}_{f}^{pl}}L\sigma_{y}d{\bar{\varepsilon }}^{pl}=\int_{0}^{{\bar{u}}_{f}^{pl}}\sigma_{y}d{\bar{u}}^{pl}$$

where ${\bar{\epsilon }}_{f}^{pl}$ is the equivalent plastic strain at failure related to the characteristic element length and ${\bar{u}}^{pl}$ is the corresponding plastic displacement. Therefore, there is no need for a lot of mesh convergence study [62,63,64].

**Figure 5:**
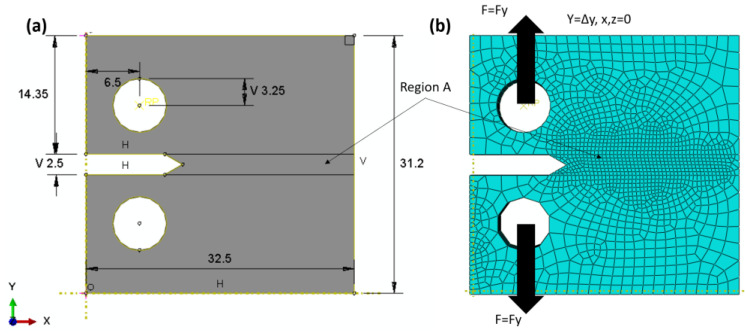
Direct cycle fatigue XFEM; (**a**) domain, (**b**) mesh and B. C domain.

The XFEM for direct cycle fatigue uses linear and nonlinear models to compare the two models, with a compact tensile test section shown with the geometry in Figure 5a. The maxps or maximum tensile separation stress was 600.5 for the Ti-27Nb alloy in the case of the linear model, while 874.63 was used for the nonlinear model and plastic flow (see Figure 1a). In addition, the damage assessment law used in the fatigue crack growth simulation was a linear independent mode, and energy types were used. The fracture energy used was 25 kJ/m^2^, as predicted in the previous sections. The element type was an eight-node linear brick with reduced integration and hourglass control (C3D8R). The total number of elements was 7560, with region A having a denser and finer mesh (see Figure 5b). The boundary condition was controlled using the maximum peak load of 3030 N, allowing one degree of freedom through the *y*-axis while the other direction was fixed. The length before the crack was 1.3 mm.

A nonlinear model with the same permeable characteristic was used for the central notch stress, and the area is the same as in Figure 3, with an initial central crack of 10 mm. The objective of using this specimen type is to study the effects of specimen types on crack growth rate. The boundary conditions were controlled strains, implementing one degree of freedom with a maximum displacement corresponding to the peak load in the y-direction, while the other directions were constrained.

To reduce the number of cycles to determine the pre-constant of the Paris equations c and m, a simple centre notch specimen with all previous linear model features was implemented in an extended finite element model with general static step.

**Figure 6:**
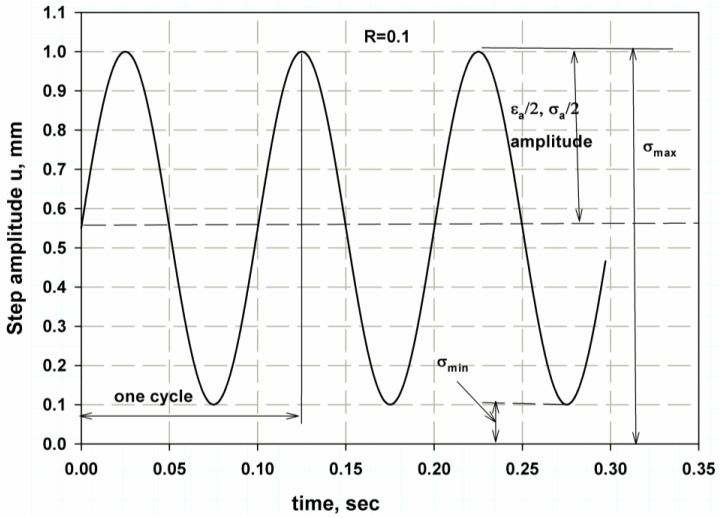
Step amplitude for fatigue analysis.

The models all require a step amplitude to generate the fluctuating load. The sine wave step shape was used with a frequency of 10 Hz. It is a fully reversed cyclic periodic step (see Figure 6) with voltage ratio and amplitude ($R=0.1,{\;\mu }_{a}=0.45$). The step amplitude form is as follows [65]:

(18) $${\;\mu }_{a}=A_{0}+\overset{N}{\underset{n=1}{\sum }}\left[A_{n}\cos n\omega \left(t-t_{0}\right)+B_{n}\sin n\omega \left(t-t_{0}\right)\right]$$

where the sin wave is $A_{n}=0$, the initial time shift is ($t_{0}=0$), and $A_{0}=0$ and $A_{1}$ = 0.45 for *N* = 1. The (ω=2πf) frequency is termed in rad/s, and the (f) frequency is termed in Hz.

## 5. Results and Discussion

### 5.1. Facture Toughness

**Figure 7:**
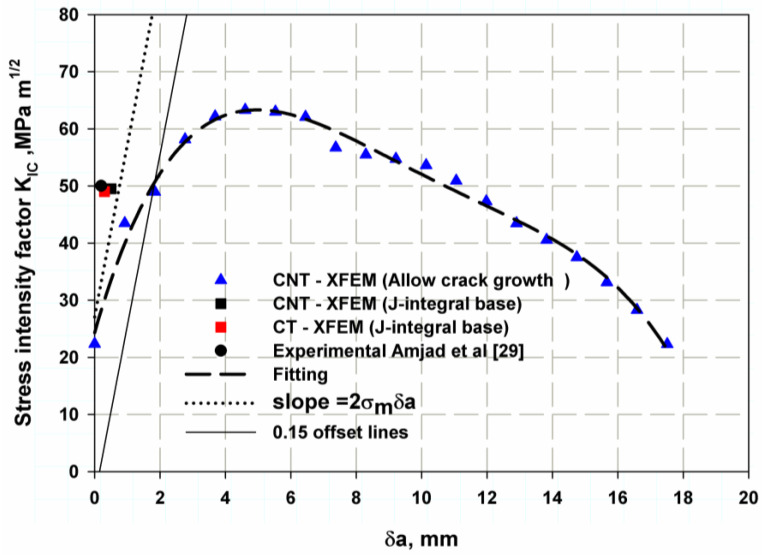
R-curve prediction using nonlinear XFEM.

**Figure 8:**
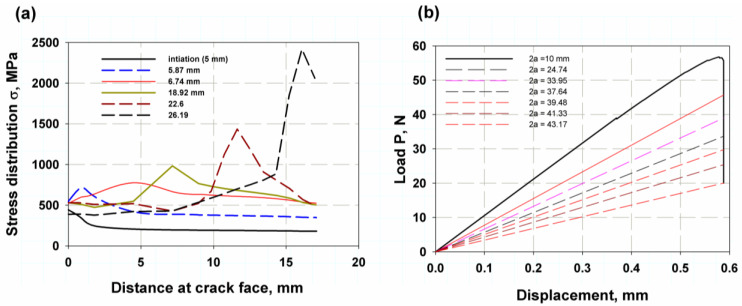
Distribution of (**a**) stress over crack, (**b**) load after crack initiation.

**Figure 9:**
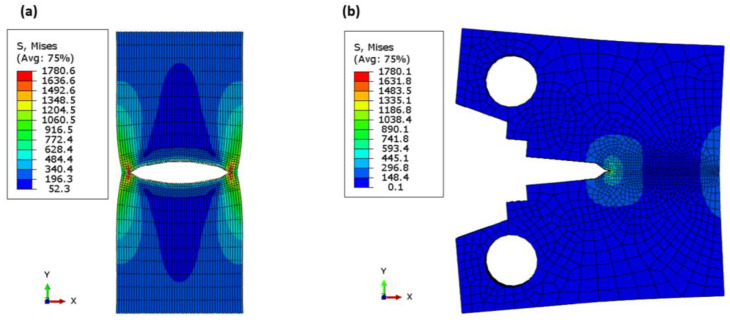
Mises contour for (**a**) XFEM, nonlinear; (**b**) XFEM (fixed crack), linear.

The Ti-27Nb alloy has a large plastic zone, so the 5% secant method proposed by the ASTM E399 standard is totally inappropriate for calculating the fracture toughness [66] since this method contains an intrinsic quantity that depends on the theoretical toughness. Therefore, the fracture toughness is preferably calculated using the J-integral method or the displacement of the crack opening. It is recommended to obtain the total R-curve. The central notch test recommended by Newman and Haines [43] was the simplest method to obtain the R-curve of ductile metal, so it was used to obtain the R-curve shown in Figure 7 (blue triangles). The initiation fracture toughness ($K_{IC}$) can be determined as reported by Kobayashi [67], and it is the intersection of the parallel line with the truncating straight lines at a crack extension of 0.15 mm. It was the point of (48.9 $\mathrm{MPa}\sqrt{m}$), and the corresponding fracture release energy $G_{IC}$ is 24.88 kJ/m^2^ These values are very close when compared with the experimental data from Ref. [29]; the percentage errors for XFEM are 2.2% and 5.2%, respectively. Therefore, the R-curve is a good simulation of the material response. The slope of the truncating straight line can be extracted from Equation (19), as follows:

(19) $$\mathrm{slop}=\gamma =2\sigma_{f}\delta a$$

where $\sigma_{f}$ is the yield stress, which is the average value between yield strength and ultimate strength, and δa is the crack length, which is 0.02 under static loading and 0.03 under dynamic loading [68]. The effect of the specimen shape and material behaviour is also illustrated in Figure 7. The single-point J-integral method FE (XFEM fixed-crack method) for the elastic or linear behaviour of a tensile specimen with a central notch and a controlled load at the point of first failure of 30,000 N (see Figure 8) for a central crack of 15 mm gives relatively similar values to the experimental data with a percentage error of 1% and 3% for the value of fracture toughness and the corresponding fracture energy, respectively. Moreover, the results of the linear or elastic model of the compact stress of a single-point FE J-integral method also provide reasonable and good results with a small percentage error of 2 and 4.5% for $K_{IC}$ and $G_{IC}$, respectively. The results of the elastic model with different specimen shapes are acceptable and in agreement with the elastic–plastic model of XFEM and the model reported in [66]. Since the different specimen shape has little effect on the fracture toughness of the material fracture across microvoids, the fracture initiation value is the value at the first crack before the material involved plastic deformation zone. Table 2 shows the comparison between the model and the available experimental results. The stress distribution over the crack area is shown in Figure 8a. The stress was low for the initiation crack length of 10 mm, and after the crack propagated, the stress-take fluctuation appeared as a peak point, then the stress decreased and this point shifted to the right side of the curve. The maximum value of peak stress was just before the complete fracture of the specimen. After the peak load was reached, the crack began to propagate, and as the crack advanced, the load gradually decreased, as shown in Figure 8b. The point of first crack was observed at the buckling point or knee for a crack length of 10 mm at a load of about 39 kN. The last length, 43.17 was not the full length of the specimen (45 mm) because the specimen in XFEM did not separate completely. The Mises stress distribution over the specimen’s surfaces showed the stress concentration zone at the crack tip in red in both models (see Figure 9a,b).

### 5.2. Direct Cycle Fatigue

**Figure 10:**
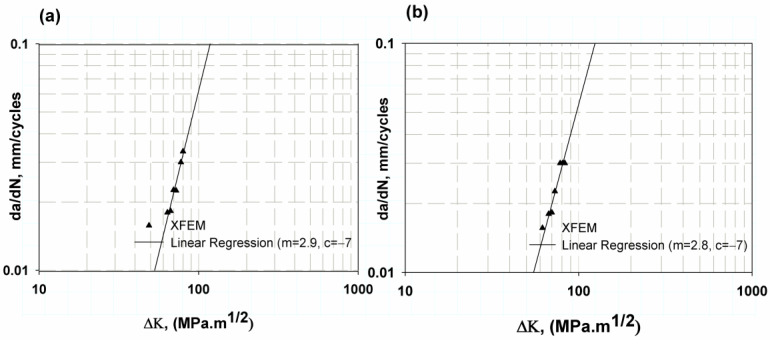
Fatigue crack growth rate for CNT (**a**) non-direct cycle fatigue, (**b**) direct cycle fatigue.

Figure 10 shows the growth of fatigue cracks for a specimen with a centre notch extracted using non-direct XFEM (static step with amplitude) and direct XFEM cycle fatigue. The log–logarithmic scale of the results was used to measure Paris’ law constants c and m. The results of the two methods are close to each other, which is due to the fact that the fracture criterion implemented in the direct cycle fatigue model uses the values of $c_{3}$ and $c_{4}$ calculated with the values of c and m predicted in the non-direct XFEM. The maximum average difference was 13.8 and 12, respectively, for the two models compared to the experimental results of Ref. [29], as listed in Table 2. This difference is accepted for fatigue results with large data points [62,69]. A centre notch tension specimen was used to determine the $c_{3}$ and $c_{4}$ components of the fracture criterion. The minimum value of dynamic fracture toughness change, ∆K, for the mean notch was 67.1 $\mathrm{MPa}\sqrt{m}$ and 63.7961 $\mathrm{MPa}\sqrt{m}$, predicted in the two models with little change due to the change in crack length in each model being a factor change in each model. The change in crack length was small. Therefore, the corresponding calculated fracture toughness in Equation (4) was small.

**Figure 11:**
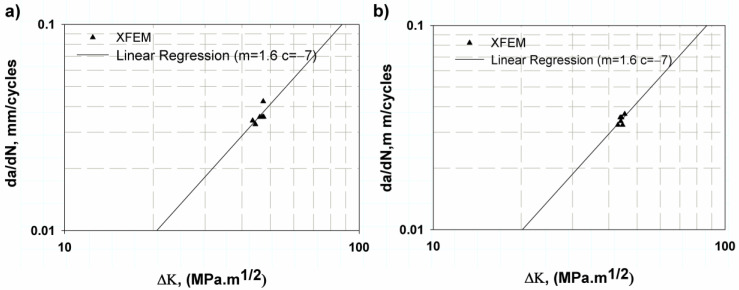
Fatigue crack growth rate for CT (**a**) Elastic behaviour, (**b**) elastic–plastic.

Figure 11 shows the Paris’ law fatigue crack growth curve for two other specimen shapes according to the ASTM standard [42] (CT) by the XFE model with direct cycle and logarithmic scale. It was found that both the elastic or linear and elastic–plastic models gave approximately the same fatigue constants, c and m (1 × 10^−7^) and 1.6, respectively (see Table 3). The percentage difference between the predicted and experimental data is relatively small, 9.2%. In addition, the predicted values for the fatigue of the large data points were within the standard ranges for the Ti-27Nb alloy; m ranges from 1.015 [9] to 3.17 [13] or 5.96 [19]. Therefore, the predicted values were accepted.

**Figure 12:**
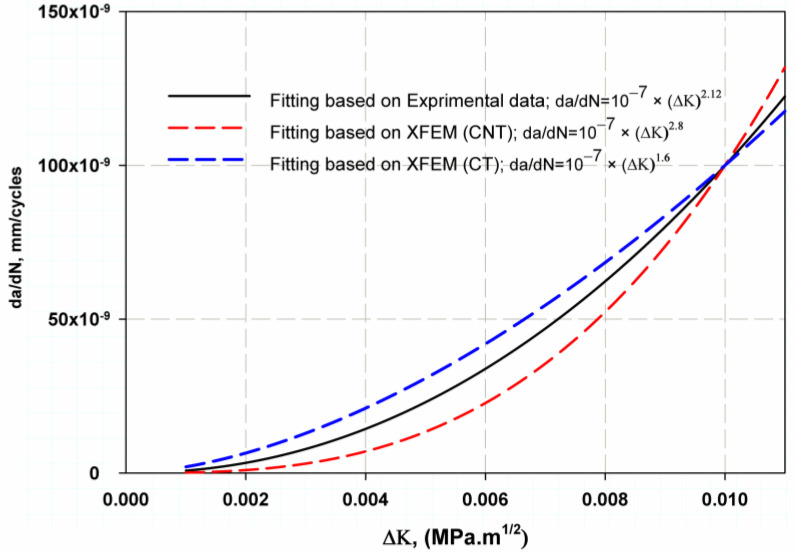
Comparison fatigue crack growth rate constant with experimental [29] results.

**Figure 13:**
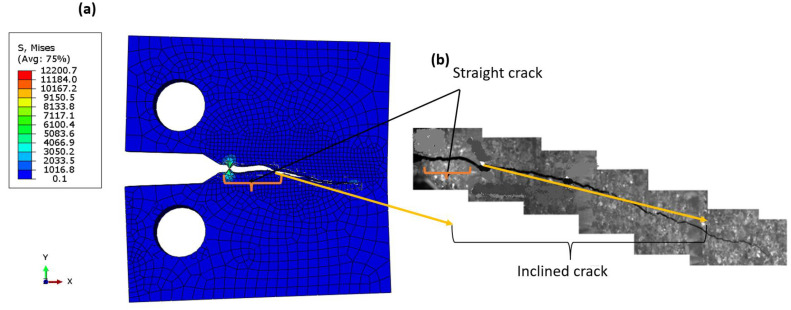
Fracture mode (**a**) XFEM (elastic), (**b**) experimental image taken from Ref. [70].

**Figure 14:**
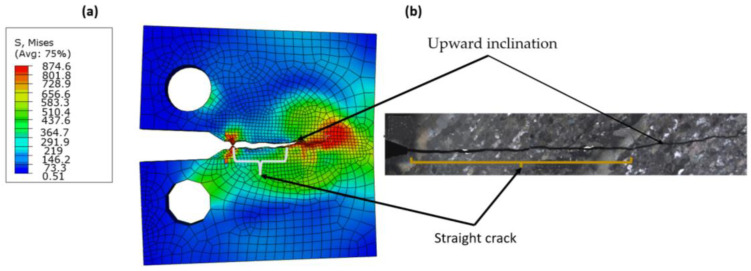
Fracture mode (**a**) XFEM (plastic), (**b**) experimental image taken Ref. [70].

Figure 12 shows the relationship between fatigue crack growth and the change in fracture toughness, ∆K, as predicted by Paris’ equation on a linear scale. It was found that the curves are close and there is little difference between the XFEM results and the experimental equation. However, the predicted values with CT stress were closer than those of CNT, and this was because the experimental data used the CT; therefore, there was a symmetry between numerical XFEM data. The fracture surface was well simulated in both models. For the elastic model (Figure 13a), the crack through two areas was straight, then inclined downward, and was almost smooth as shown by the experimental work of Ref. [70], which was due to the anisotropic structure of Ti-27Nb alloy in this orientation [70]. In the plastic model, the crack was shorter (Figure 14a) than the experimental image of Ref. [70]. The crack surface was not smooth but had a lamellar microstructure [13].

## 6. Conclusions

Fracture toughness was predicted for alloy Ti-27Nb as an attractive biomaterial used extensively for human implants using a full R-curve method for a standard notch stress specimen. The XFEM accuracy was less than 2.2% and 5.2% for the corresponding fracture energy G_IC_. The model types and specimen shape for anisotropic materials such as Ti-27Nb have little effect because the measured fracture toughness was the initiation fracture toughness, which is related to the point at which the crack propagates, commonly known as the peak load point. The fatigue crack growth rate da/dN was well predicted and its relationship with the change in fracture toughness ∆K, known as Paris’ law, was recognised; the equation constant c and m were predicted with a minimum percent difference of less than nine. In addition, the type of specimen and material behaviour has a discernible effect on fatigue crack growth. Fatigue fracture modes for Ti-27Nb were well predicted, as two crack paths were identified that led to complete fracture with a laminar surface crack.

## Figures and Tables

**Table 1:** Mechanical properties of Ti-27Nb alloy [29].

Properties	Value
Density (Kg/m^3^)	4520
Young’s modulus (GPa)	87.45
Yield strength (MPa)	600.5
Ultimate strength (MPa)	874.63
Fracture strain	0.0264571
Strain rate, (mm/mm)/s	0.166
Poisson’s ratio, µ	0.33
N, strain hardening	0.7
K, (MPa)	1148

**Table 2:** Comparison between the different FE models with experimental results.

Models	Experimental [29]	XFEM	%Error	J-Integral (Fixed-Crack XFEM)			
				CNT	%Error	CT	%Error
Fracture toughness K_IC_, MPam^1/2^	50	48.9	2.2	49.5	1	49	2
Release energy G_IC_, kJ/m^2^	26.3	24.8	5.2	25.5	3	25	4.5

**Table 3:** Fatigue crack growth data comparison of Ti-27Nb alloy.

FEM Model	Fatigue Constants		%Average Difference
	m	c	
Experimental data [29]	2.12	$10^{-7}$	
CNT–non-direct cycle XFEM–elastic model	2.9	$10^{-7}$	13.8
CNT–direct cycle XFEM–elastic model	2.8	$10^{-7}$	12
CT-elastic XFEM	1.6	$10^{-7}$	9.2
CT–elastic–plastic (nonlinear behaviour) XFEM	1.6	$10^{-7}$	9.2

## Data Availability

The data presented in this study are available on request from the corresponding author.
